# Molecular Docking Compounds of Cinnamaldehyde Derivatives as Anticancer Agents

**DOI:** 10.31557/APJCP.2021.22.8.2409

**Published:** 2021-08

**Authors:** Warsito Warsito, Shinta Murlistyarini, Suratmo Suratmo, Vina O Azzahra, Andrian Sucahyo

**Affiliations:** 1 *Faculty of Mathematic and Natural Sciences, Essential Oil Institute, Brawijaya University, Malang, Indonesia. *; 2 *Laboratory of Biomedic, Faculty of Medicine, Brawijaya University, Malang, Indonesia. *

**Keywords:** Anticancer, cinnamaldehyde, hydroxy cinnamaldehyde, methoxy cinnamaldehydes, molecular docking

## Abstract

**Objective:**

Cinnamaldehyde (CM) has a molecular structure with the main reaction center of an aromatic ring which the bioactivity can be modified as an anticancer agent by substituting the groups in the ortho (o), meta (m), and para (p) position. The present study aimed to investigate the correlation of the cluster region that was substituted in CM on its activity for various anticancer receptors.

**Methods:**

The receptor types used in the test were 5FL6, 1HOV, 4GY7, 5EAM, 4XCU, 4EL9, and 4PQW. The suitability of the hydroxy (OH) and methoxy (OMe) groups, which were substituted, was studied based on the value of Ki, their interactions with metal cofactors, and the type of amino acid residues that function as cancer receptor inhibitors. The docking was conducted using AutoDock 4.

**Results:**

The study results showed that all derivative compounds (o, m, and p) –OH and –OMe CM commonly had better anticancer activities than CM. o-OH CM has the best activity against receptors 5FL6, 1HOV, 4GY7, 5EAM, and 4XCU, and m-OMe CM has better activity against the 4EL9 receptors when compared with other CM derivatives.

**Conclusion:**

Based on this study, the compound derived from CM, i.e. OHC, tends to show the best anticancer activity.

## Introduction

Quantitative structure–activity relationship (QSAR) and molecular docking contribute significantly to the rationale designing of novel drug discovery (Sunyoung et al., 2019; Roberts et al., 2020). QSAR provides a role in modern chemistry by describing a molecular activity, with statistical analysis in silico, to simplify and reduce in vivo testing. However, QSAR is considered less popular, and the results are not obtained as fast as those of molecular docking (Santiago et al., 2008; Sunyoung et al., 2019; Roberts et al., 2020). Over the last few years, molecular modeling has provided the latest results in studying the main structural requirements, geometric modeling approaches, and analysis of the interaction between ligands and receptors by molecular docking (Hanine and Menana, 2020). Some of the applications used in molecular docking for decades include AutoDock, FlexX, Surflex, Glide, LigandFit, Autodock Vina, rDock, and UCSF Dock. Among these applications, AutoDock is one of the most popular applications and one of those having a top-ranking performance with the best score (Nataraj et al., 2007).

Cinnamaldehyde (CM) is an interesting drug development base because it can be obtained from synthesis and isolation of natural ingredients. CM is the main component of cinnamon bark essential oil, which has been widely used as a herbal medicine, especially in tropical and subtropical areas (Ha-Won et al., 2003; Nor and Ngadiwiyanan, 2006; Dimas and Koen, 2017). It demonstrates as an antiallergenic, antimicrobial, antiviral, antioxidant, gastroprotective agent, and anti-Alzheimer’s, which has attracted the attention of several research studies (Nor and Ngadiwiyanan, 2006; Yulius, 2013; Su-Hyung et al., 2016). Additionally, CM has antiangiogenetic properties among which being an active compound used as an antitumour and anticancer agent (Kuswandi et al., 2016).

CM was studied for its use as an anticancer agent, which can inhibit apoptosis of liver cancer cells (hepatocellular carcinomas [HCCs]) by interfering with the CD95/CD95L-CASP-8 signal flow in anomalous cells type p53 (HepG2) (Liang-Tzung et al., 2013), inhibit the proliferation of human metastatic melanoma cell type (A375, G361, and LOX) in vitro in the G1 phase cell cycle (Peter et al., 2010), and induce apoptosis of blood cancer cells (leukemia) K562 (Yonika and Edy, 2018). The activity of CM as an anticancer and chemopreventive agent indicates the potential of CM to be developed as an anticancer agent in various cancer types.

CM’s unique basic framework is also of value in developing compound derivatives. CM has two electrophilic reaction sites: β-carbon in the conjugated double bond and carbon from the aldehyde carbonyl group. Previous studies have suggested that the bioactivity of CM and its derivatives are influenced by the potential of Michael acceptors and the ability of nucleophilic attacks at the β-carbon site (Amanda and John, 2019). The conjugated double bond of the phenyl group is worth studying for its derivatives because it can be substituted in the ortho, meta, and para positions to enhance its activity as an anticancer agent.

The addition of functional groups contained in CM makes it possible to have some interesting CM derivatives to study. The addition of hydroxy and methoxy groups to the phenyl group that acts as an antioxidant agent reduces the level of oxidative stress that resulted in a chronic disease; these unique structural properties are potential anticancer agents (Shahriar and Robin, 2010). The addition of hydroxy groups to CM derivatives, namely, orto-hydroxy CM (OHC), meta-hydroxy CM (MHC), and para-hydroxy CM (PHC), and methoxy derivatives, namely, orto-methoxy CM (OMC), meta-methoxehyde (OMC), and para-methoxyCM (PMC) is interesting to be tested for their potential activities on various cancer receptors.

## Materials and Methods

*Sample collection*

The information of CM derivatives in the present study was obtained from the literature (Ha-Won et al., 2003; Su-Hyung et al., 2016), and the structures of these ligands were downloaded from www.pubchem.ncbi.nlm.nih.gov. The macromolecules of target comprised anticancer receptors, and their structures were downloaded from www.rcsb.org including 1HOV, 4GY7, 5EAM, 4XCU, 4EL9, 4PQW, and 6PGX.

*Molecular docking analysis*

All of CM derivatives’ three-dimensional data in .SDF format were converted to .pdb using the Discovery Studio 2019 application. All receptors were optimized by removing water, HetAtm, and leaving Chain A and metal ions that act as catalysts. Then, the receptors and ligands were superimposed in the Discovery Studio 2019 application. Furthermore, docking was conducted using the AutoDockTools 1.5.6 application for each ligand with a receptor where the docking stage was conducted by running Autogrid and then running Autodock (Syed et al., 2013). The setting of the grid box size was adjusted to the active side of each receptor. The charge of receptors that have metal ions were optimized to +2 by changing their notion manually in .pdb format using notepad. One of the 10 conformations results from the docking was selected for re-docking with the same grid box size setting as the first docking until the RMSD value was <2 Å.

## Results

*Human Carbonic Anhydrase IX (hCA IX) (various cancers)*

**Figure 1:**
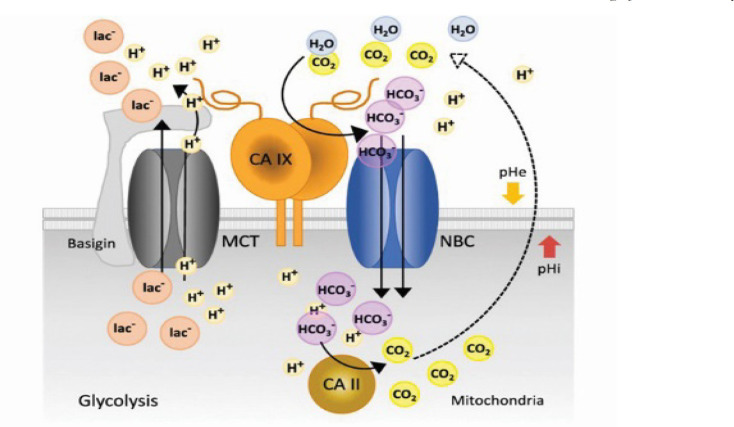
Schematic Representation of the Role of CA IX on pH Regulation in Hypoxic Cancer Cells (Silvia and Roberts, 2019).

Protein carbonic anhydrase (CA) has a Zn^2+^ metal active side that is coordinated with the imidazole rings of three histidine residues and one hydroxide ion from water. CA catalyzes the reversible hydration of CO_2_ to bicarbonate and a proton. In a cell, bicarbonate interacts with intracellular protons results of several metabolic mechanisms. This pathway (possibly catalyzed by the cytoplasmic isoform of CA II) leads to the conversion of bicarbonate to CO_2_. The consumption of intracellular protons from the presence of bicarbonate ions helped increase the intracellular pH to a permissive value for metabolic processes, signaling, and proliferation. Conversely, the extracellular protons resulted from CA IX catalytic reaction remained outside of the cell and contributed to the acidification of the pericellular milieu (Figure 1). Excessive extracellular acidification may promote the invasion of cancer cells into surrounding normal tissue (Mam et al., 2018).

In the condition of hypoxic tumour cells, the pH of the extracellular environment (pHe) is lower than in normal conditions (pHe ≥ 7.3) of around 6.5–7.1. Because of the lower pHe conditions, the acid-prone environment supports the survival and proliferation of tumour cells (Mam et al., 2018).

**Figure 2:**
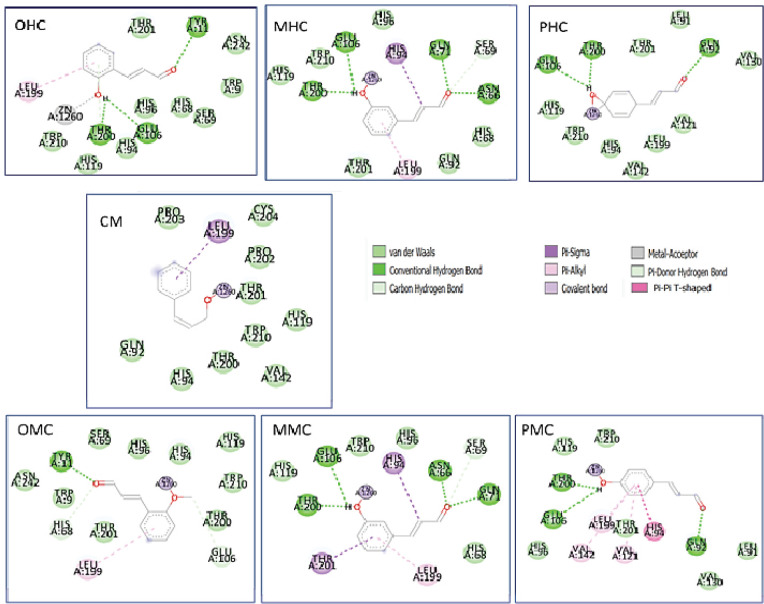
Two-Dimensional Interaction of the Cinnamaldehyde Compounds and Their Derivatives against the Receptor hCA IX

As shown in Table 1, the CM compounds and all CM derivatives interacted with Zn^2+^ metal by covalent bonds (Figure 2). In the CM compounds, the observed interactions were the π-sigma between phenyl and Leu 199, and the covalent bond between Zn metal and aldehyde. The interaction of CM was weaker than the derivative compounds shown with the highest Ki value of 363.47 µM. OHC demonstrates the lowest Ki value among ligands, occupying the strongest interaction with the receptors compared to other compounds, and this interaction is supported by the interaction of metal acceptors with Zn metal. The compounds with the best potential when sorted by Ki value are OHC > OMC > MMC > MHC > PMC> PHC > CM.

*Matrix Metalloproteinase-2 (various cancers)*

The active site of matrix metalloproteinase-2 (MMP-2) comprises zinc metal and consensus (HEBGHXLGLXHS) of amino acids for the Zn-binding motif, which are conserved throughout MMP-2; three histidine residues are bound to Zn (Santiago et al., 2008). Several previous studies have shown that amino acids in the catalytic cavity play an active role in the binding of an inhibitor to the receptor protein cavity. This affects MMP-2 activity in the breakdown of the extracellular matrix in normal processes in tissues, as well as in metastases and arthritis (Masantos et al., 2006; Xian-Chao et al., 2008 Jose et al., 2011). The inhibitor can provide activity if it can target Zn metal as an essential part, as well as the S1′ specificity loop in MMP-2, with an important region between Tyr142 and Leu150 (Yiqing et al., 2002).

**Figure 3:**
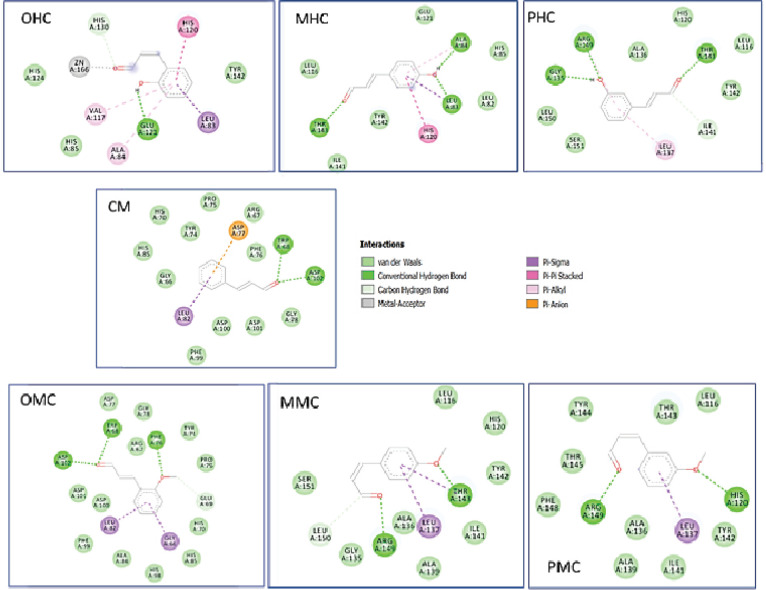
Two-Dimensional Interaction of Cinnamaldehyde Compounds and Their Derivatives against the Receptor MMP-2

The results showed that OHC is the potential compound as an inhibitor of MMP-2 because it directly interacted with Zn metal (Table 2). This compound also interacted with the Tyr142 of the S1′ pocket with van der Waals interaction. Additionally, PMC showed the ability to interact with six residues, the highest amount as compared with other ligands, within S1 important region, suggesting that is has a good activity as an inhibitor. The results showed that CM and OMC compounds could not interact with the Zn metal or S1 region. Although the compounds that have the potential to be an MMP-2 inhibitor were perceived from the Ki score and the number of interactions with the S1′ pocket, the list of candidates sorted from the best is as follows: PMC > MMC > PHC > MHC > OHC. Therefore, OHC compounds were perceived more as potential anticancer agents as compared with other CM compounds (Figure 3).

*Stomach cancer (urease)*

Urease (urea amidohydrolases) is a contributor that induces the massive survival of *Helicobacter pylori*. The active side of the urease enzyme lies in the nickel metal (Ni^2+^) and the sulfhydryl group, where the active site plays a role in the catalytic effect of the urease enzyme. Urease is responsible for the hydrolysis of urea resulting ammonia. Ammonia will neutralize the acid produced in the stomach resulting in a pH environment conducive to the survival and colonization of *H. pylori* bacteria (Muhammad et al., 2018). *H. pylori* infection can cause chronic inflammation and significantly increase the risk of developing duodenal ulcer disease and gastric cancer. In addition, the infection by H. pylori is the most potent known risk factor for developing gastric cancer (Roberts et al., 2018).

The Cys592 function (denoted by the name CME 592) is the primary residue and has a position on the mobile flap covering the active site. This residue of Cys592 becomes an essential control in positioning other significant residues in the active site for catalytic purposes. This modification of CME 592 causes difficulty in the flap movement that keeps the flow there active, interrupting further reactions and reducing the activity of the urease enzyme (Lirong et al., 2013).

**Figure 4:**
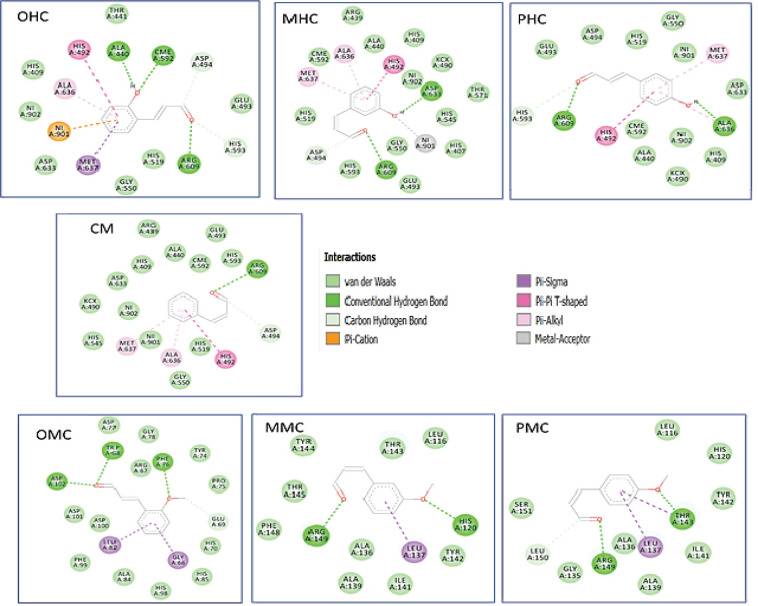
Two-Dimensional Interaction of the Cinnamaldehyde Compounds and Their Derivatives against the Urease Receptor

The results showed that OHC compounds interacted with Ni^2+^ metal by pication and also interacted with the thiol group CME 592 by hydrogen bonding (Table 3 and Figure 4). Therefore, it was perceived that the OHC compound could act as an inhibitor of the urease enzyme, which can cause stomach cancer. Conversely, the CM compounds could not interact with Ni2+ metal and only interacted with CME 592 by van der Waals forces, so their interactions are not as strong as the hydrogen bonds in OHC compounds. As for OMC, MMC, and PMC, the results showed that they could not interact with both Ni metal and CME592. Meanwhile, compounds that can potentially act as urease inhibitors based on Ki and their interaction with an essential part of the active site are OHC > MHC > PHC.

*Blood cancer (WDR5)*

WD repeat-containing protein 5 (WDR5) is an essential component of the multiprotein complex that can activate mixed-lineage leukaemia (MLL1). WDR5 binds to MLL1, which has a critical role in the stability and methyl-transferase activity of the MLL1 complex (MLL1, RbBP5, WDR5, and DPY-30). Therefore, the deterioration of either one of these complexes will significantly reduce or eliminate the catalytic activity of this complex. In addition, WDR5 has been shown to facilitate the regulation of H3K4 trimethylation by MLL1 via binding to the N-terminal of histone H3 (Getlik et al., 2016).

The interaction between MLL1-WDR5 occurs via a WIN motif located in the N-terminal ~50 amino acids of the SET region, where Arg3765 (in MLL1) has an essential role in making contact with WDR5. The mutation of Arg3765 significantly reduces the stability of the MLL1 complex and abolishes the dimethyltransferase activity of MLL1 H3K4 (Anamika et al., 2008).

**Figure 5:**
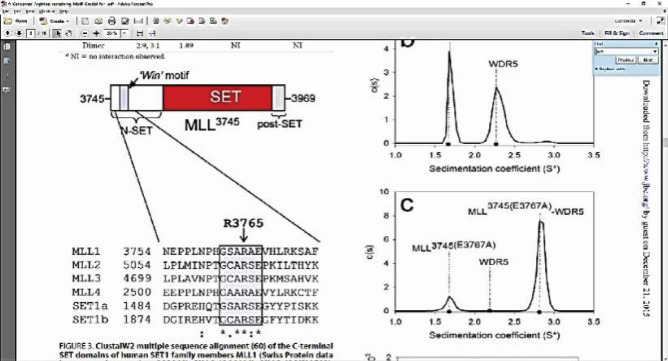
Sequence Alignment of the C-terminal SET Region of Human SET1 Members of the MLL1 Family (Anamika et al., 2008).

As shown in Figure 5, the amino acid GSARAE in MLL1 includes the Arg3765 region essential for interacting with WDR5. The crystal structure of WDR4 binds to the peptide WIN motif of MLL1, indicating that the Arg-3765 of the MLL1 WIN motif binds to the central arginine-binding pocket of WDR5 (Anamika et al., 2008). The amino acids Ser91 and Phe133 are located on the surface of the central arginine-binding pocket of WDR5. When Ser91 is replaced with lysine, it is expected to interfere with the entry of the arginine side of Arg3765 into the binding pocket. Phe133 makes hydrophobic and π-cation interactions with guanidinium arginine and has previously been shown to be essential for the interaction of histone H3 with WDR5. When Ser91 and Phe133 were mutated, the two proteins could not interact with Arg3765 from MLL1 (Anamika et al., 2008).

Arg3765 from MLL1 also enters the center hole of WDR5 and is stabilized by the presence of hydrogen bonds, π-π and cation-π, and hydrophobic interactions with the following WDR5 residues: Ser91, Phe133, Ser175, Ser218, Cys261, Phe263, and Ile305 (Ji-Joon and Roberts, 2008).

**Figure 6:**
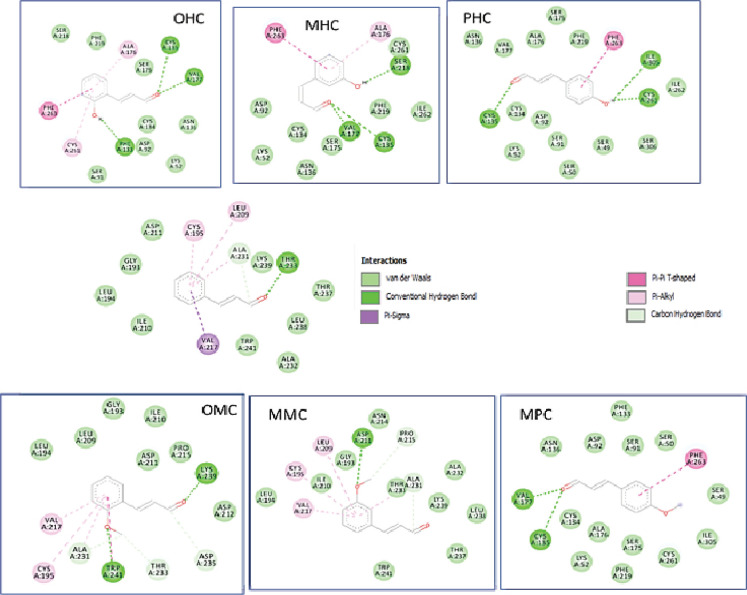
Two-Dimensional Interaction of the Cinnamaldehyde Compounds and Their Derivatives against WDR5

As shown by the docking results in Table 4, the compounds that have the potential to act as inhibitors of WDR5 were OHC, MHC, PHC, and PMC because of their activities against several major amino acids, such as Ser91, Phe133, Ser175, Ser218, Cys261, Phe263, and Ile305 (Figure 6). These compounds are bound in the central arginine-binding pocket of WDR5, where the interaction between WDR5 and MLL1 occurs. OHC showed the greatest potential since it interacted with six amino acids in the central arginine-binding pocket and had a strong bond with Phe133 by hydrogen bonding, Cys261 on a pi-alkyl basis and Phe263 in Pi-Pi T-shaped. Conversely, CM, OMC, and MMC were suggested to have no potential activities against the main amino acids.

*Liver cancer (HCCs)*

Fibroblast growth factor 19 (FGF19) is a hormone that regulates bile acid synthesis and hepatocyte proliferation in the normal liver through the activation of its receptor, that is, fibroblast growth factor receptor 4 (FGFR4). Bile acids induce the expression of FGF19 in the intestine and increase the circulating hormone level, thereby causing hepatic repression of cholesterol 7α-hydroxylase (CYP7A1) and mediating an essential stage in hepatic bile acid synthesis. It has been studied previously that the overexpression of FGF19 in transgenic mice can produce liver tumours that are sensitive to the treatment with FGF19 or FGFR4 antagonist antibodies. Additionally, genetic defects against FGFR4 prevented transgenic mice from growing tumours. Therefore, we need a compound that can target FGFR4 to treat patients with HCC (Margit et al., 2015).

The compound tested and acted as a strong inhibitor of FGFR4 is BLU9931, which binds covalently to the ATP-binding pocket of FGFR4, the amino acid Cys552. Therefore, a compound that can be covalently bonded with the amino acid Cys552 is designed (Margit et al., 2015).

**Figure 7:**
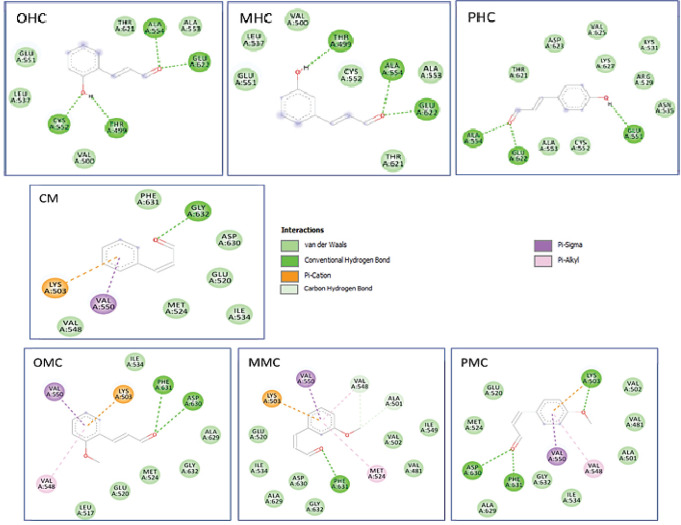
Two-Dimensional Interaction of the Cinnamaldehyde Compounds and Their Derivatives against FGFR4

None of the cinnamaldehyde compounds and their derivatives interacted covalently with Cys552, which makes them less potential as inhibitors of FGFR4 (Table 5 and Figure 7). The OHC compounds only interact with Cys552 hydrogen bonds, whereas MHC and PHC have only weakly interacted through van der Waals force with Cys552. Meanwhile, CM, OMC, PMC, and MMC have no potential as inhibitors of FGFR4 for HCC.

*RSK2 (various cancer)*

Protein phosphorylation is one of the main regulatory mechanisms in all eukaryotic cells. Humans have 518 protein kinases that catalyze the activity of protein target by phosphorylation of the Ser/Thr or Tyr residues. One of the new families of Ser/Thr protein kinases recognized as potential targets by a drug is the p90 ribosomal s6 (RSK) kinase. The known RSK isoform is RSK1-4 has a C-terminal kinase domain and a physiologically active N-terminal kinase domain. RSK1 and RSK2 have become the main focus of researchers regarding their association with various cancers.

The compound that has been tested as an inhibitor of RSK2 is SL0101. The amino acid residues that bind to SL0101 are Ile50 and Ile52; when the two amino acid residues were mutated, they could no longer bind to RSK2. Additionally, the amino acid Phe79 residue is also essential for the expression of kinase activity; this is because Phe79 protects the triphosphate group from ATP and the substrate phosphorylation site from the solvent. When Phe79 was mutated into mutant F79A, this mutant became resistant to SL0101 (Darkhan et al., 2012).

**Figure 8:**
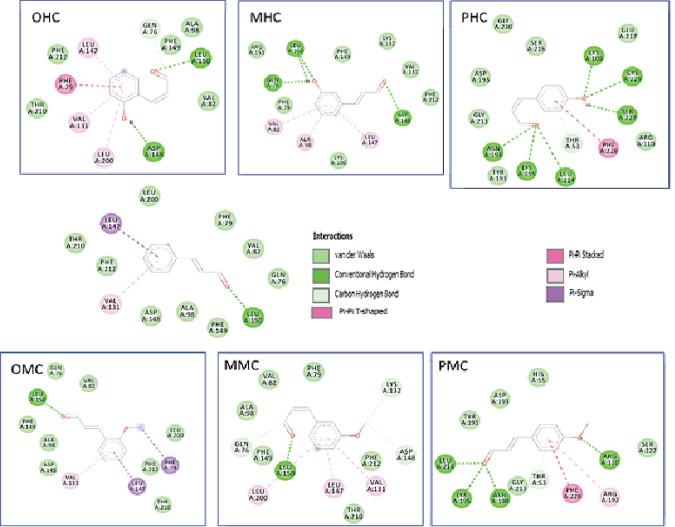
Two-Dimensional Interaction of the Cinnamaldehyde Compounds and Their Derivatives against RSK2

As shown in Table 6, no compounds interact with Ile50 and Ile52, the essential amino acid residues that interact with the SL0101 inhibitor. As for OHC, PHC and PMC did not interact with the main residues of Ile50, Ile52, or Phe79. Compounds that may have the potential to act as inhibitors of RSK2 were OMCs, which interacted with Phe79 using Pi-sigma. Conversely, MMC and MHC compound only interacted with van der Waals force against Phe79 (Figure 8).

*Pancreatic cancer (NHERF1)*

CXC chemokine receptor 2 (CXCR2) is a receptor linked to a G protein activated by binding to the chemokines Gro-α, Gro-β, Gro-γ, ENA-78, GCP-2, IL-8, or NAP-2. CXCR2 mediates neutrophilic migration and plays an essential role in positioning oligodendrocyte precursors in spinal cord development. These receptors also function in angiogenesis and wound healing and contribute to spontaneous tumourigenesis and that induced by inflammation. CXCR2 signalling promotes the progression of pancreatic cancer, where its increased expression correlates with the aggressive stage in the patient.

The formation of the macromolecular complex of CXCR2-NHERF1-PLCβ3 in pancreatic cancer cells regulates the signalling activity of CXCR2 and plays an essential role in tumour proliferation and invasion. Hence, we looked for an inhibitor of NHERF1 that can block the interaction between NHERF1-PLCβ3. These inhibitors can inhibit pancreatic tumour growth by reducing signaling from CXCR2, which prevents tumour cell proliferation and tumour invasion.

The interaction between NHERF1-PLCβ3 occurs at hydrophobic sites where the amino acid residues that participate in the interaction are Tyr24, Phe26, Leu28, Val76, and Ile79. There are also hydrogen bonds with Phe26, Tyr24, and Gly25. Other residues that took part in the interaction were His27 (van der Waals), His72 (hydrogen bonds), Leu28 (hydrogen bonds), Arg40 (hydrogen bonds), and dan Gly30 (Yuanyuan et al., 2014).

**Figure 9:**
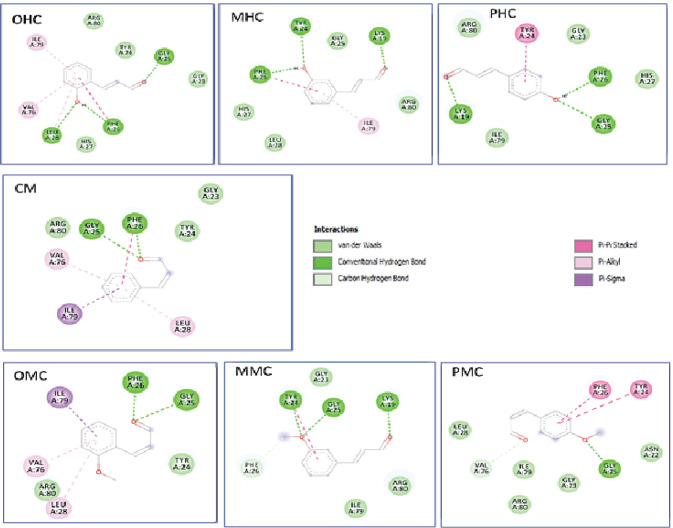
Two-Dimensional Interaction of the Cinnamaldehyde Compounds and Their Derivatives against NHERF1

Based on the docking results in Table 7, CM had the smallest Ki value of −5.49, and this compound interacted with six essential amino acid residues from NHERF1. The CM compound itself already has potential as an inhibitor of NHERF1 when viewed via an in silico approach. However, the efficacy of this compound should be compared via an in vitro approach as its derivatives such as OMC, PMC, OHC, MHC, and PHC were also interacted with the six essential residues but had different types of interactions (Figure 9). 

**Table 1:** Docking Results of the Compound Cinnamaldehyde and Its Derivatives to hCA IX (ID: 5FL6)

Compound	Ki (µM)	Gibbs energy (kcal/mol)	RMSD (Å)	Interaction Zn2+
Cinnamaldehyde (CM)	363.47	−4.69	0.14	√
o-methoxy cinnamaldehyde (OMC)	61.91	−5.74	0.14	√
m-methoxy cinnamaldehyde (MMC)	65.68	−5.71	0.36	√
p-methoxy cinnamaldehyde (PMC)	83.58	−5.56	0.32	√
o-hydroxy cinnamaldehyde (OHC)	31.15	−6.15	0.23	√
m-hydroxy cinnamaldehyde (MHC)	66.96	−5.69	0.2	√
p-hydroxy cinnamaldehyde (PHC)	84.99	−5.55	1.12	√

**Table 2:** Docking Results of the Compound Cinnamaldehyde and Its Derivatives to MMP-2 (ID: 1HOV)

Compound	Ki (µM)	Gibbs energy (kcal/mol)	RMSD (Å)	Interaction Zn2+	Interaction (142–150)
CM	23.56	−6.31	0.56	–	–
OMC	11.40	−6.74	0.84	–	–
MMC	79.32	−5.59	0.49	–	4
PMC	77.22	−5.61	0.59	–	6
OHC	91.42	−5.51	1.77	√	1
MHC	83.27	−5.56	0.29	–	2
PHC	108.95	−5.41	0.48	–	4

**Table 3:** Docking Data Results of the Cinnamaldehyde Compounds and Their Derivatives against the Urease Receptor (ID: 4GY7).

Compound	Ki (µM)	Gibbs energy (kcal/mol)	RMSD (Å)	Interaction Zn2+	Interaction CME592
CM	112.54	−5.39	0.15	–	√
OMC	265.42	−4.88	0.19	–	–
MMC	354.44	−4.71	0.19	–	–
PMC	515.74	−4.49	0.13	–	–
OHC	186.00	−5.09	0.44	√	√*
MHC	78.56	−5.60	0.20	√	√
PHC	328.56	−4.75	0.28	√	√

*, higher interaction with the corresponding amino acid

**Table 4:** Docking Results Data from the Cinnamaldehyde Compounds and Their Derivatives against WDR5 (ID: 5EAM).

Compound	Ki (µM)	Gibbs energy (kcal/mol)	RMSD (Å)	Interactions with amino acids						
				Ser91	Phe133	Ser175	Ser218	Cys261	Phe263	Ile305
CM	95.16	−5.49	0.65	–	–	–	–	–	–	–
OHC	64.00	−5.72	1.73	√	√*	√	√	√*	√*	–
MHC	37.00	−6.04	0.15	–	–	√	√*	√	√*	–
PHC	69.70	−5.67	0.27	√	–	√	–	√*	√*	√*
OMC	18.79	−6.45	1.45	–	–	–	–	–	–	–
MMC	40.17	−6.00	0.51	–	–	–	–	–	–	–
PMC	139.1	−5.26	0.74	√	√	√	–	√	√*	√

**Table 5:** Docking Results Data of the Cinnamaldehyde Compounds and Their Derivatives against FGFR4 (ID: 4XCU).

Compound	Ki (µM)	Gibbs energy (kcal/mol)	RMSD (Å)	Interaction Cys552
CM	380.83	−4.66	0.58	–
OHC	718.19	−4.29	1.89	√*
MHC	661.64	−4.34	0.07	√
PHC	532.4	−4.47	0.12	√
OMC	144.14	−5.24	0.19	–

**Table 6:** Docking Results Data from the Cinnamaldehyde Compounds and Their Derivatives against RSK2 (ID: 4EL9).

Compound	Ki (µM)	Gibbs Energy (kcal/mol)	RMSD (Å)	Interaction Ile50	Interaction Ile52	Interaction Phe79
CM	95.26	−5.49	0.71	–	–	√
OHC	44.71	−5.93	0.21	–	–	–
MHC	34.81	−6.08	0.16	–	–	√
PHC	17.72	−6.48	1.36	–	–	–
OMC	61.56	−5.74	0.86	–	–	√*
MMC	54.50	−5.82	1.85	–	–	√
PMC	39.11	−6.01	0.65	–	–	–

**Table 7:** Data from Docking Results of the Cinnamaldehyde Compounds and Their Derivatives against NHERF1 (ID: 4PQW).

Compound	Ki (µM)	Gibbs Energy (kcal/mol)	RMSD (Å)	Interaction									
				Tyr24	Phe26	Leu28	Val76	Ile79	Gly25	His27	His72	Arg40	Gly30
CM	95.26	−5.49	0.71	√	√*	√*	√*	√*	√*	–	–	–	–
OMC	170.71	−5.14	0.29	√	√*	√*	√*	√*	√*	–	–	–	–
MMC	162.43	−5.17	0.31	√*	√	–	–	√	√*	–	–	–	–
PMC	351.21	−4.71	1.80	√*	√*	√	√*	√	√*	–	–	–	–
OHC	201.17	−5.04	0.54	√	√*	√*	–	√*	√*	√	–	–	–
MHC	109.75	−5.40	0.69	√*	√*	√	–	√*	√	√	–	–	–
PHC	164.2	−5.16	0.55	√*	√*	–	–	√	√*	√	–	–	–

*, higher interaction with the corresponding amino acid

## Discussion

Various cancers that are used as receptors are hCA IX, MMP-2, and RSK2, which have general mechanisms of action in the body. As a group of various cancers, the act of hCA is not specific because the reaction mechanism that occurs outside the cell affects the pHe of the cell environment. All CM ligands and their derivative compounds interact with Zn^2+^ metal by covalent bonds, except for OHC compounds with metal-acceptors. Based on molecular docking results, all compounds used as ligands are potential inhibitors of hCA IX. Inhibitor action can interfere with the hCA IX-catalyzed reaction by catalyzing the conversion of bicarbonate to CO_2_, which causes an increase in intracellular acidity and facilitates the invasion of cancer cells so that the presence of CM ligands and their derivatives inhibits the metabolism and proliferation of cancer cells, with OHC and CM as the most and least potential compounds, respectively.

MMP-2, which is used as a receptor, acts on the cellular system. CM and its derivative compounds gave a distinct type of interaction to the amino acid Tyr142 – Leu150 of MMP-2. Meanwhile, CM and OMC have no potential as inhibitors compared with the other five compounds. Our results showed that OHC and PMC were suggested as the most potential compounds for interfering MMP-2, because the former can interact with Zn and Tyr 142. The latter can interact with six amino acids in the S1′ pocket of MMP-2. Meanwhile, the CM compounds and their derivatives showed no significant potential for various cancers of RSK2; of these compounds, OHC showed the best result.

The specific cancer types used in the present study were gastric cancer (urease), blood cancer (WDR5), HCCs, and pancreatic cancer (NHERF1). Urease induces the suitable habitat of *H. pylori*, a species that is one of the main causes of gastric cancer. An essential part in the active side that controls enzyme catalysis is the nickel-metal and sulfhydryl groups, and on the CME 592 of the active side flap. OHC interacted with CME 592 with hydrogen bonds, indicating urease inhibition activity in hydrolyzing urea. Other compounds such as MHC and PHC have a potential activity as inhibitors but with less possibility than OHC.

WDR5 plays an essential role in protein expression in MLL1 blood cancer. Various primary amino acids influence its catalytic activity. OHC, MHC, PHC, and PMC compounds can be potential inhibitors. The activity level of these compounds should be determined via in vitro testing, depending on the amino acid that has the most influence on the inhibitory bonding activity between WDR5 and MLL1. The other receptor is HCC. In the mechanism of action of HCC liver cancer, FGFR19 plays an essential role in treatment because it can regulate bile synthesis. The seven compounds did not show sufficient potential for activity. The OHC tends to have an activity as an HCC inhibitor as compared with the other six compounds.

In pancreatic cancer receptors, CM has the best activity value as an inhibitor of the formation of the CXCR2-NHERF1-PLCβ3 complex in pancreatic cancer cells. The activity of each CM ligand and its derivatives was different between the main amino acids Tyr24, Phe26, Leu28, Val7, Ile79, Gly25, and His27, although all of the seven compounds did not interact with His72, Arg40, and Gly30. Their activity should be tested to determine the effect of specific amino acids on the activity of the ligands.

Based on the studies and the results of molecular docking, it can be concluded that with the receptors of hCA IX, MMP-2, gastric cancer, blood cancer WDR5, and HCC, the compound derived from CM, that is, OHC, tends to show the best activity. In various cancers that are associated with RSK2, OMC has the best activity. CM tends to have the best activity as compared with its derivatives against pancreatic cancer receptors. The results of this molecular docking study serve as an introduction to future research. The present study should be followed by an in vitro investigation to determine the activity of compounds used in this study.

## Author Contribution Statement

The authors confirm contribution to the paper as follows: study conception and design : Warsito Warsito; data collection: Vina O Azzahra, Andrian Sucahyo; analysis and interpretation of results: Shinta Murlistyarini, Suratmo; draft manuscript preparation: Warsito Warsito, Vina O Azzahra. All authors reviewed and approved the final version of the manuscript.

## References

[B1] Amanda D, John CS (2019). A review of cinnamaldehyde and its derivatives as antibacterial agents. Fitoterapia.

[B2] Anamika P, Venkatasubramanian D, Michael SC (2008). Structure of WDR5 bound to mixed lineage leukemia protein-1 peptide. J Biol Chem.

[B3] Darkhan U, Urszula D, Natalya O (2012). nsights into the inhibition of the p90 ribosomal S6 kinase (RSK) by the flavonol glycoside SL0101 from the 1.5 Å crystal structure of the N-terminal domain of RSK2 with bound inhibitor. Biochemistry.

[B4] Dimas RAM, Koen D (2017). Cinnamon and its derivatives as potential ingredient in functional food—A review. Int J Food Prop.

[B5] Getlik M, Smil D, Zepeda V (2016). Structure-based optimization of a small molecule antagonist of the interaction between WD repeat-containing protein 5 (WDR5) and mixed-lineage leukemia 1 (MLL1). J Med Chem.

[B6] Hanine H, Menana E (2020). 2D and 3D-QSAR, molecular docking and ADMET properties: In silico studies of azaaurones as antimalarial agents. New J Chem.

[B7] Ha-Won J, Dong H, Kwang-Hee S, Mi-Young H (2003). Antitumour effect of the cinnamaldehyde derivative CB403 through the arrest of cell cycle progression in the G 2 / M phase. Biochem Pharmacol.

[B8] Ji-Joon S, Robert EK (2008). WDR5 interacts with mixed lineage leukemia (MLL) protein via the histone H3-binding pocket. J Biol Chem.

[B9] Jose MZ, Pilar S, Josune GS (2011). Potent ‘clicked’ MMP2 inhibitors: Synthesis, molecular modeling and biological exploration. Org Biomol Chem.

[B10] Kuswandi M, Kusumaningtyas E, Santoso B (2016). Sintesis Senyawa (2E)-3-Fenilprop-2-Enoil 3,4,5- Trihidroksibenzoat dengan menggunakan microwave [Synthesis of compounds (2E) -3-Phenylprop-2-Enoil 3,4,5- Trihydroxybenzoate using microwave]. 4th Univesity Res Coloquium.

[B11] Liang-Tzung L, Shu-Jing W, Chun-Ching L (2013). The anticancer properties and apoptosis-inducing mechanisms of cinnamaldehyde and the herbal prescription Huang-Lian-Jie-Du-Tang (Huáng Lián Jiě Dú Tang) in human hepatoma cells. J Tradit Complement Med.

[B12] Lirong T, Jiyan S, Dianwei W (2013). Kinetics and mechanism study of competitive inhibition of Jack-Bean urease by baicalin. Sci World J.

[B13] Mam YM, Brian PM, Robert M, Susan CF (2018). Carbonic anhydrases: Role in pH control and cancer. Metabolites.

[B14] Margit H, Chandra M, Michael S (2015). First selective small molecule inhibitor of FGFR4 for the treatment of hepatocellular carcinomas with an activated FGFR4 signaling pathway. Cancer Discov.

[B15] Masantos AS, Sergio MM, Tiziano T (2006). Design, synthesis and molecular modeling study of iminodiacetyl monohydroxamic acid derivatives as MMP inhibitors. Bioorganic Med Chem.

[B16] Muhammad A, Muhammad O, Mamoon UR (2018). NMR, novel pharmacological and in silico docking studies of oxyacanthine and tetrandrine: bisbenzylisoquinoline alkaloids isolated from Berberis glaucocarpa roots. J Anal Methods Chem.

[B17] Nataraj SP, Khajamohiddin S, Jack T (2007). Software for molecular docking: a review. Biophys Rev.

[B18] Nor BAP, Ngadiwiyanan N (2006). Identifikasi senyawa penyusun minyak kulit batang kayu manis (Cinnamomum cassia) menggunakan GC-MS [Identification of cinnamon bark oil compounds (Cinnamomum cassia) using GC-MS]. J Kim Sains dan Apl.

[B19] Peter L, Vanessa Zl, Jennie C (2010). Host-cell sensors for Plasmodium activate innate immunity against liver-stage infection. Nat Med.

[B20] Robert S, Malgorzata S, Jerzy M 2018) Gastric cancer: epidemiology, prevention, classification, and treatment. Cancer Manag Res.

[B21] Robert A, Bogdan T, Cristina SS, Mihaela D (2020). Use of QSAR global models and molecular docking for developing new inhibitors of C-src tyrosine kinase. Int J Mol Sci.

[B22] Santiago V, Giorgio C, Stefano M (2008). Medicinal chemistry and the molecular operating environment (MOE): Application of QSAR and molecular docking to drug discovery. Curr Top Med Chem.

[B23] Shahriar K, Robin JM (2010). Monocyclic phenolic acids; hydroxy- and polyhydroxybenzoic acids: Occurrence and recent bioactivity studies. Molecules.

[B24] Silvia P, Roberts JG (2019). The role of carbonic anhydrase IX in cancer development: links to hypoxia, acidosis, and beyond. Cancer Metastasis Rev.

[B25] Sunyoung K, Ho B, Jeonghee J, Sungroh Y (2019). Comprehensive ensemble in QSAR prediction for drug discovery. BMC Bioinformatics.

[B26] Su-Hyung H, Ismail AI, Sung-Min K, Dong CH, Byoung-Mog K (2016). Cinnamaldehydes in cancer chemotherapy. Phyther Res.

[B27] Syed MDR, Shazi S, Mohd H (2013). A simple click by click protocol to perform docking. Excli J.

[B28] Xian-Chao C, Qian W, Hao F, Wei T, Wen-Fang X (2008). Design, synthesis and preliminary evaluation of novel pyrrolidine derivatives as matrix metalloproteinase inhibitors. Eur J Med Chem.

[B29] Yiqing F, John JL, Leiming Z, Harold W (2002). Solution structure and backbone dynamics of the catalytic domain of matrix metalloproteinase-2 complexed with a hydroxamic acid inhibitor. Biochim Biophys Acta - Protein Struct Mol Enzymol.

[B30] Yonika AL, Edy M (2018). Revealing the potency of cinnamon as an anti-cancer and chemopreventive agent. Indones J Cancer Chemoprevention.

[B31] Yuanyuan J, Shuo W, Joshua H (2014). Crystallographic analysis of NHERF1-PLCβ3 interaction provides structural basis for CXCR2 signaling in pancreatic cancer. Biochem Biophys Res Commun.

[B32] Yulius F (2013). Prospek pengembangan kayu manis (Cinamon Burmanii L) di Indonesia [Prospect of cinnamon development (Cinamon Burmanii L) in Indonesia]. Sirinov.

